# Wellcome’s Photographic Exposure

**Published:** 1908-01

**Authors:** 


### Wellcome's Photographic Exposure.

SO strong in these strenuous days, is the demand upon time and labor that, in every field of endeavor, devices and contrivances for the saving of both have found ready acceptance though, in accordance with past experience, many of them prove to be short lived or fall by the wayside. In no work is more good technic demanded throughout than in photography, and no sphere has the various products of ingenuity enabled the amateur to avail himself of the experience of the professional.

In no part of photographic work has the professional, as well as the amateur, been more perplexed or felt the need of something, than in the keeping of systematic records of exposures. Many charts, exposure records, and the like, have been brought out, but few have outlived their birth or filled the indication.

We have carefully looked over "*Wellcome's Photographic Exposure Record and Diary*" published by Burrough's Wellcome & Company, London and New York, and, in comparing its adaptation to the wants of those for whom it is intended, it can be truly said that it covers the ground in a more complete and satisfactory manner than anyother yet brought out. It covers, in a concise way, a most extensive field, taking special note of such features where open questions are involved, and gives decisions and recommendations with reasons for the same, which are as valuable as cogent.

It includes a consideration of developers, papers, toning, and goes largely into the question of timing, with simplified rules, applicable to all conditions and circumstances, and in such a way as to simplify this difficult part of the work and make available the results of experience and study, to such an extent as to make the amateur not only feel confident, but the professional secure. It contains forms for hundreds of exposure records, a dairy for every day in the year, and a calculator that is so simple and yet efficient as to satisfy the most exacting.

Space will not permit a detailed account of the range of its application. We can, though, without hesitation, say that with its aid anyone—amateur or professional—could carry on his work under all circumstances without any other reference. It is unusually well gotten up, being substantial, attractive, and includes the best printing, paper and workmanship. It represents an enormous amount of labor, and is a working companion for the photographer who, once possessed of it, would part with it with the greatest reluctance. It is compact and can be carried in the vest pocket; and, to sum up, nothing could be more appropriate than its name—"*Wellcome.*"

